# Localisation tubaire et ovarienne d'une malakoplakie: à propos d'un cas et revue de la literature

**DOI:** 10.11604/pamj.2015.22.17.6561

**Published:** 2015-09-09

**Authors:** Ikram Boubess, Salma Ouassour, Mokha Tazi, Adib Filali, Mohammad Alami

**Affiliations:** 1 Service de Gynécologie-Obstétrique Maternité des Orangers CHU Ibn Sina, Rabat, Maroc

**Keywords:** Corps de Michaelis-Gutmann, granulomatose inflammatoire, kyste ovarien suspect, Michaelis-Gutmann bodies, inflammatory granulomatosis, suspicious ovarian cyst

## Abstract

La malakoplakie est une pathologie inflammatoire rare qui résulte d'un déficit de la fonction phagocytaire macrophagique. Il n'existe pas de symptomatologie spécifique de la maladie mais dépendante de l'organe touché. Nous rapportons un cas rare de malakoplakie annexielle chez une femme de 36 ans qui a été opérée pour suspicion de cancer ovarien et dont le diagnostic de malakoplakie ne s'est fait qu’à l'examen anatomopathologique. La malakoplakie touche essentiellement le tractus urogénital et le diagnostic positif repose seulement sur l'histologie. Le traitement est basé sur l'antibiothérapie et l'exérèse chirurgicale si mauvais état de l'organe atteint.

## Introduction

La malakoplakie est une granulomatose inflammatoire rare liée à un déficit de la phagocytose macrophagique, définie histologiquement par l'accumulation intracellulaire de fragments bactériens appelés corps de Michaelis-Gutmann. La malakoplakie est une affection bénigne et rare fréquente au niveau du tractus génito-urinaire. Elle n'a aucune particularité clinique ou paraclinique et le diagnostic est histologique. L'origine infectieuse associée a une atteinte locale de la fonction des macrophages est la théorie physiopathologique la plus soutenue [1]. Le traitement est surtout médical et l'exérèse chirurgicale n'est indiquée qu'en cas de destruction totale de l'organe atteint. Nous rapportons le cas d'une patiente qui a été opérée pour suspicion de tumeur ovarienne maligne et dont l'examen anatomopathologique était en faveur d'une malakoplakie.

## Patient et observation

**Figure 1:**
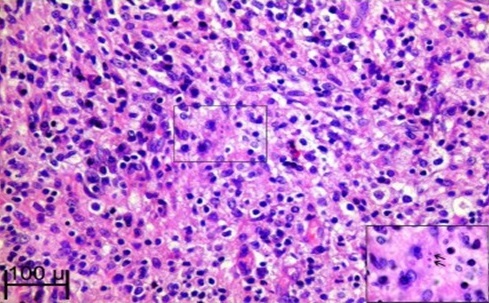
Lésion pathognomonique de la malakoplakie-corps de Michaelis et Gutmann conférant aux histiocytes l'aspect en cible ou en œil d'oiseau; HE Grossissement x 20

N.F est une patiente de 36 ans, sans antécédents pathologiques notables, troisième geste troisième pare avec trois enfants vivants accouchés tous par césarienne, qui a consulté à la maternité des orangers pour douleurs pelviennes chroniques sans signes associées notamment pas de notion d'infection urinaire à répétition. L'examen clinique a trouvé une patiente en bonne état général avec à l'examen gynécologique la découverte d'une masse latéro-utérine rénitente et douloureuse à la mobilisation. L’échographie chez cette patiente a confirmé la présence d'une masse latéro-utérine droite suspecte qui pouvait être en rapport avec l'ovaire droit. La tomodensitométrie a confirmé la présence d'un kyste ovarien d'allure malin avec suspicion d'envahissement colique et d'une carcinose péritonéale. L'exploration chirurgicale a mis en évidence une masse inflammatoire de l'annexe droite avec présence des plaques jaunâtres sur la trompe et l'ovaire concernés sans signes de malignité macroscopiques avec agglutination des anses intestinales autour de l'annexe. Le traitement s'est limité à une annexectomie droite vu le mauvais état de la trompe et de l'ovaire. L'examen anatomopathologique de la pièce reséqué était en faveur d'une malakoplakie en localisation annexielle (Figure 1).

## Discussion

Le terme malakoplakie est dérivé du mot latin: malako (soft) et plakie (plaque) [1]. C'est une pathologie rare décrite pour la première fois par Michaelis et Guetmann et une centaine de cas qui a été publié dans la littérature depuis sa description en 1902. L'atteinte extra-urinaire n'a été décrite qu’à partir de 1958 [2]. Cette maladie s'observe à tout âge avec un pic de fréquence aux alentours de la cinquantaine mais on a rapporté des cas d'atteinte aux âges extrêmes. En cas de localisation urinaire isolée, la femme est plus touchée que l'homme mais pour les autres atteintes extra-urinaires les hommes sont les plus touchés [3]. Toutes localisations confondues, les deux sexes sont également atteints [4]. Les localisations urogénitales sont les plus fréquentes, elles se voient dans 58% des cas [5]. Aucun signe clinique n'est spécifique de cette pathologie car la symptomatologie est liée à l'organe atteint. L'atteinte génitale féminine, est très rare [6], la revue de la littérature a relevé seulement quelques cas de localisations ovariennes [7, 8] similaires à notre cas clinique avec suspicion de tumeur ovarien maligne et le diagnostic de la malakoplakie n'a pas été fait qu'après l'examen anatomopathologique de la pièce opératoire. Dans notre cas la symptomatologie était dominée par les douleurs pelviennes et l'aspect macroscopique des lésions peut conclure à une forme pseudotumorale de la malakoplakie.

L'apport de la biologie est dominé par un syndrome inflammatoire non spécifique, l'apport de l'imagerie médicale est limité pour faire le diagnostic de la malakoplakie [9]. Sur le plan anatomopathologique la malakoplakie réalise un granulome qui est caractérisé par la présence des cellules de Van Hansemann [10]. Ces cellules sont des histiocytes à larges cytoplasmes, riches en granulations éosinophiles et des enclaves basophiles de grande taille contenant les corps de Michaelis et Guetmann (CMG). Ces CMG confèrent aux histiocytes l'aspect en cible ou d'un œil d'oiseau et constituent la lésion pathognomonique de la malakoplakie. Les progrès réalisés dans la connaissance de la physiopathologie de la malakoplakie ont rendu cette affection accessible au traitement médical. Ce traitement consiste essentiellement sur l'association d'un antibiotique le plus souvent basé sur les quinolones, qui ont montrés une efficacité thérapeutique par rapport a la Sulfaméthoxazole/Triméthoprime qui était le plus utilisé, associé à un cholinergique [11, 12], l'adjonction de l'acide ascorbique peut performer le traitement selon certains auteurs [13]. Un traitement au long cours doit être instauré pour éviter les récidives et les extensions aux autres organes. L'exérèse chirurgicale n'est indiquée qu'en cas de lésion sévère de l'organe atteint ce qui est le cas chez notre patiente.

## Conclusion

La malakoplakie est une inflammation granulomatose tissulaire d'aspect histologique caractérisé par la présence de corps de Michaelis et Guetmann. Les formes cliniques sont variées mais sa forme pseudo tumoral est trompeuse. Le traitement est basé sur une antibiothérapie adaptée sauf si lésion organique sévère où le traitement chirurgical est indiqué.
